# Lifestyle and behavioural changes in older adults during the Covid-19 pandemic are associated with subjective cognitive complaints

**DOI:** 10.1038/s41598-024-52856-0

**Published:** 2024-01-30

**Authors:** Janos Andras Zsuffa, Sandor Katz, Vanda Koszovacz, Dalida Borbala Berente, Anita Kamondi, Gabor Csukly, Francesca Mangialasche, Ana Sabsil Lopez Rocha, Miia Kivipelto, Andras Attila Horvath

**Affiliations:** 1 Department of Family Medicine, Semmelweis University, 9 Stahly utca, Budapest, 1085 Hungary; 2 Neurocognitive Research Center, National Institute of Mental Health, Neurology and Neurosurgery, Budapest, Hungary; 3 Department of Anatomy Histology and Embryology, Semmelweis University, Budapest, Hungary; 4 Department of Psychiatry and Psychotherapy, Semmelweis University, Budapest, Hungary; 5 School of PhD Studies, Semmelweis University, Budapest, Hungary; 6 Department of Neurology, Semmelweis University, Budapest, Hungary; 7 Division of Clinical Geriatrics, Department of Neurobiology, Care Sciences and Society, Center for Alzheimer Research, Karolinska Institutet, Stockholm, Sweden; 8 Medical Unit Aging, Theme Inflammation and Aging, Karolinska University Hospital, Stockholm, Sweden; 9 The Ageing Epidemiology Research Unit, School of Public Health, Imperial College London, London, UK; 10 Institute of Public Health and Clinical Nutrition, University of Eastern Finland, Kuopio, Finland

**Keywords:** Risk factors, Neurological disorders

## Abstract

Subjective cognitive complaints (SCC) is a self-reported experience of persistently impaired cognitive functions which could be the earliest red flag of neurocognitive disorders. The COVID-19 pandemic and related restriction measures changed the lifestyle and behaviour of older adults. The aim of this study was to assess the relation of these changes and SCC status in Hungary. This cross-sectional study analysed the data of 359 elderly Hungarians who filled out the WW-FINGERS-SARS-CoV2 survey. A quarter of the respondents (n:88) reported SCC in connection with the pandemic. We compared sociodemographic features, health status, lifestyle, and social life parameters between subjects with reported SCC and without. To eliminate the potential interrelation across group differences, stepwise logistic regression was applied. Participants with SCC showed the following characteristics, compared to individuals without: (1) they were older; (2) they were more likely to be women; (3) they had a higher number of chronic disorders; (4) showed more prominent impairment in physical mobility; (5) had worse sleep quality; (6) spent less time with family; and (7) used internet more frequently during the pandemic (all *p*’s < 0.001). Logistic regression highlighted that only two parameters were related to SCC status independently, the physical mobility (ability to walk 500 m without difficulties; OR = 1.186; *p* < 0.001; 95%CI = 1.101, 1.270) and changes in time spent with grandchildren (OR = 1.04; *p* = 0.015; 95%CI = 1.008, 1.073). Our study draws attention to the importance of physical mobility and quality time with family as key factors in the cognitive well-being of elderly people.

## Introduction

With an aging population cognitive impairment due to major neurocognitive disorders such as Alzheimer’s disease (AD) is a growing public health concern. According to the estimations, over 55 million people are living with AD and other dementias worldwide, and this number is expected to reach 139 million by 2050^1^. Dementia is characterized by cognitive and functional impairment that leads to loss of daily functioning and independence^2^. It is often preceded by mild cognitive impairment (MCI), which is an early stage of cognitive decline in individuals who maintain the ability to independently perform most activities of daily living. This condition is also characterised by objective cognitive deterioration^3^.

Subjective cognitive complaints (SCC) represent a self-reported experience of persistently impaired cognitive functions (e.g., memory, visuo-spatial skills, language functions). The condition is an integral component of the diagnostic criteria of MCI^4^ and also a key hallmark of subjective cognitive decline (SCD), where individuals show a normal performance on standardised cognitive tests^5^. The utility of SCC in the prediction of cognitive decline shows ambiguous results in a short-interval period due to (1) the possible overreporting of SCC in individuals with higher level of anxiety or mood problems^6^, or (2) the anosognosia/reduced awareness of their cognitive symptoms in patients with neurocognitive disorders^7^. However, a large meta-analysis of 28 studies^8^ demonstrated that SCC was associated with a relative risk of 2.07 for the development of dementia after 4 years follow-up. Furthermore, a hallmark longitudinal autopsy study confirmed the long-term predictive value of SCC showing that subjective complaints precede the diagnosis of MCI by over 9-years^9^. These findings have been confirmed by further studies, particularly in SCC population with chronic cardiovascular disease^10^.

In March 2020, the World Health Organization declared COVID-19 a pandemic^11^. In many countries around the world severe restrictions have been introduced during peaks of infection. To slow down the spread of infections, countries introduced general mobility restrictions, limitation of social gathering and strict infection control measures in the healthcare system^12^. In addition to the negative health consequences of COVID-19 infection, the introduction of strict quarantine and lockdown measures has disrupted social networks and damaged the global economy. As a result, there is growing concern that the pandemic has been affecting the mental health and the quality of life of the population^13^. One of the most vulnerable groups in the COVID-19 pandemic is the population over 60 years of age, as they had the highest morbidity and mortality. They often must deal with significant health and economic challenges by themselves as persons living with chronic diseases and being both isolated and with limited access or ability to use digital technology^14^.

In Hungary, the third wave of the COVID-19 pandemic began from February 2021. An increasingly significant number of illnesses were caused by mutations that spread faster than before, especially the so-called British (alpha) virus variant. The symptoms were more severe, and the daily number of new deaths linked to the coronavirus has gradually increased from below 100 in the second half of January 2021; by the end of March, it repeatedly exceeded 300 cases a day. In Hungary, the total number of deaths linked to the coronavirus increased from ten thousand to thirty thousand during the third wave, and the total number of people infected with the COVID-19 virus also doubled during this period. Radical restrictions were also introduced in Hungary: in addition to the closure of kindergartens and primary schools, non-essential shops and services were suspended, and the use of masks was required in public areas. The health sector also mainly focused on the treatment of cases related to the coronavirus and those that cannot be postponed. Mass vaccinations against COVID-19 also took place during this period; for the first time, at the end of December 2020. Healthcare workers were the first to be vaccinated, and later the oldest residents and patients with certain chronic diseases. From April 2021, in accordance with the increase in the number of vaccinated people, the restrictions were gradually eased; by the beginning of June 2021—after the number of daily new cases and deaths had significantly decreased—the end of the third wave was announced. Taking into account the previous description, the Hungarian population of older adults was also socially isolated during the pandemic, which had a significant negative impact on their lifestyle and health^15^.

In the current cross-sectional study, we aimed to analyse the social and the lifestyle changes during the third wave of the pandemic in an elderly population. Our special aim was to characterize the most relevant predisposing factors for SCC in relation of the COVID-19 pandemic and following life-style changes.

## Methods

### Participants and data acquisition

The study was conducted within the framework of the World-Wide (WW)-FINGERS network of multidomain clinical trials for dementia risk reduction (led by Prof. Miia Kivipelto, Karolinska Institute, Sweden)^16^. Within the network, the WW-FINGERS-SARS-CoV-2 initiative was launched in response to the COVID-19 pandemic, under the aegis of the World Health Organization (WHO) Neurology and COVID-19 Global Forum, to assess direct and indirect effects of the pandemic in midlife and older age^17,18^. The survey focused on changes in lifestyle factors, management of chronic noncommunicable diseases, as well as psychosocial factors, all of which are relevant to cognition and are expected to be affected by the pandemic. Local results in some countries are already available^18–20^.

In Hungary, the study was led by the consortium of the National Institute of Mental Health, Neurology and Neurosurgery and by the Department of Psychiatry and Psychotherapy, Semmelweis University. We collected data using the Hungarian translation of the „World-Wide Fingers SARS-CoV-2 Survey” (Hungarian WW-Fingers SARS CoV2 Survey) between 1st of February and 1st of June 2021, covering the third wave of the COVID-19 pandemic in Hungary (Budapest and neighbouring suburban area). Inclusion criteria were the followings: (1) age 60 + years; (2) living in Hungary; (3) being fluent in Hungarian language; (4) the absence of previous diagnosis of major neurocognitive disorders based on the available medical records. Participation was on a voluntary base and included patients from GP practices, residents of nursing homes, and residents from inpatient institutions. The above categories included healthy elderly participants and patients with various types of chronic diseases. For obtaining the data, a paper-based, self-administered questionnaire was used. Data were recorded in uniform format defined by the WW-FINGERS Network, the standardized modules allow later international comparisons. The Research Ethics Committee of the National Institute of Mental Health, Neurology and Neurosurgery authorized our ethical protocol (reference number: IKEB 17/2020). All respondents agreed to participate in the survey with their informed written consent. All methods were carried out in accordance with relevant guidelines and regulations.

In total 431 participants answered the Hungarian WW-Fingers SARS CoV2 survey. The vast majority of respondents were patients in GP practices. A small number of respondents, only 7%, (30/431) lived in a nursing homes. Since we did not aim to analyze the direct impact of COVID-19 infection, we excluded persons self-reported previous COVID-19 virus infection (n = 26). We also excluded participants having possible cognitive impairment at the time of data acquisition [existing diagnosis of minor neurocognitive disorders (n = 8) and subjects who preferred not to disclose their pandemic cognitive status (n = 4)]. Those participants who reported better subjective memory performance since the start of COVID-19 pandemic (n = 4) or subjects preferred not to report their subjective memory changes (n = 30) were also excluded.

**Figure 1:**
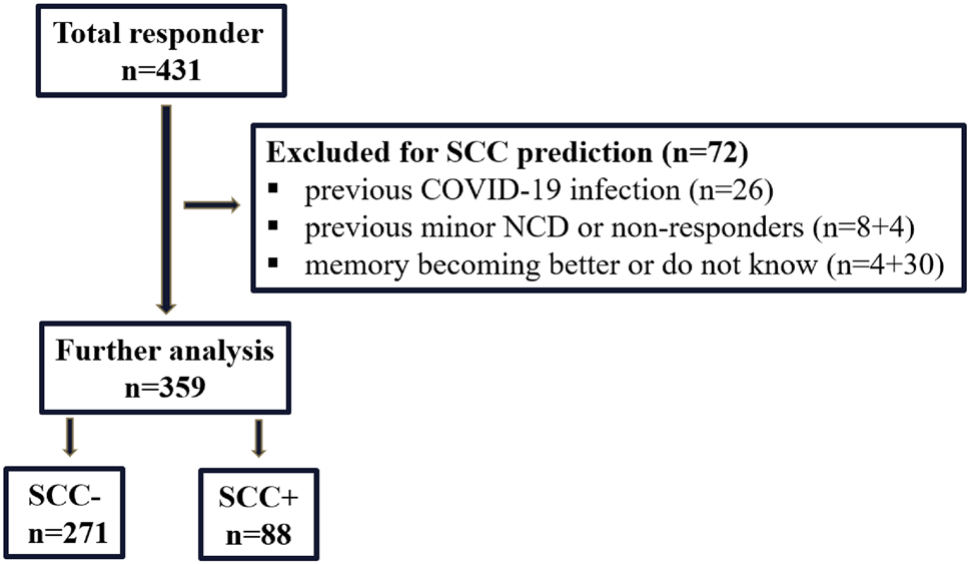
Flowchart of the participant selection for the SCC prediction. Key: COVID-19: coronavirus disease 2019, NCD: neurocognitive disorders, SCC: subjective cognitive complaints.

For statistical analysis the answers of 359 participants (age: 73.6 ± 7.9, 223 females) were processed. Those 271 (75.5%) participants who had intact cognitive performance before the pandemic and did not experience any change in their cognitive functions were included in the SCC− group. The remaining 88 (24.5%) who had normal cognitive performance preceding the pandemic but reported worsening cognitive performance (affecting mostly the memory functions) since the outbreak were selected in the SCC+ cohort. In SCC+ group 45 participants worried about the memory worsening. Figure 1 displays the flowchart of the participant selection for the SCC prediction.

### Survey

The questionnaire was built up by 46 questions, focusing on six main domains, which are presented in more detail in Table 1.

**Table 1:** Main questions of the survey.

Sociodemographic characteristics and living settings	Age, sex, educational status, marital status, administrative classification of the residence, number of people living together, age of people living together
Medical conditions and biometric data	Height, pre-pandemic and current body weight, body mass index, pre-pandemic diagnoses of chronic physical and mental diseases, physical mobility
Parameters related to COVID-19 infection	Symptoms, diagnosis, screening method, treatment and care, activities to reduce the spread of the pandemic, vaccination status, rate and duration of physical and social isolation
The direct and indirect effects of the pandemic on lifestyle and behaviour	Smoking, alcohol consumption, physical activity, social contacts, sleeping habits, eating habits, digital device use, remote work, internet and media use
Changes in the access to health care services at the time of the pandemic	Number of needed medical visits, cancellation rate, reasons for delayed visits, services related to emergency care, dental treatment, mental health care, social assistance, home care
Social engagement before and during the pandemic	Time spent with family members/friends, volunteering, charity, education, sport/social clubs, organizations

### Data extraction and statistical analysis

From 4 question groups, 5–5 parameters were selected that were consistent with the previously reported cognitive risk parameters^21–24^. As a major selection criterion, only statistically independent parameters were included for further analysis, where the risk that one variable directs the changes of the others is enough low. Independence was checked with a correlation matrix where r was set as  < 0.35 in significant correlations (*p* < 0.05) or p was not significant (*p* > 0.05) (Table S1 in Supplementary Material). The thresholds are defined based on traditional medical statistical opinions^25^. From the sociodemographic category age (in years), sex (possible answers- 1: female, 2: male, 3: prefer not to say), educational attainment (in years), family status (possible answers- 1: single, 2: married, 3: living with partner, 4: in relationship, living separately, 5: divorced, 6: widowed, 7: prefer not to say) and employment status (possible answers:- 1: employed, 2: temporally unemployed due to pandemic, 3: unemployed, 4: pensioner, 5: working as a pensioner, 6: prefer not to say) were selected. From information regarding lifestyle, medical conditions and biometric data, pre-pandemic smoking status (scale- 1: no, 2: sometimes 3: daily), pre-pandemic alcohol consumption (scale- 1: 1–2 international unit (IU), 2: 3–4 IU, 3: 5–6 IU, 4: 7–9 IU, 5: > 10 IU/day), pre-pandemic body mass index (BMI), current number of chronic disorders and physical independence measured as the capability of independent walking of 500 m (possible answers- 1: easily able, 2: able but with difficulties, 3: barely able, 4: not able) were selected. From the lifestyle changes, the followings were selected measured with a 5-point scale (1: significantly decreased, 2: decreased, 3: same, 4: increased, 5: significantly increased): time spent with family, time spent doing physical activity, presence of sleep problems, time spent on remote working, time spent with internet use. From the social engagement response pool, the followings were selected measured on a 7-point scale (1: daily, 2: few times per week, 3: once per week, 4: few times per month, 5: once per month, 6: less than once per month, 7: never): time for grandchildren, time for voluntary work, time for educational activity, time for sport and social clubs, time for patient organizations. Pre- and post-pandemic responses were compared, and changes were highlighted on a scale ranging from − 6 (maximum increase) to + 6 (maximum decrease).

Distribution of the numeric variables was analysed with Kolmogorov–Smirnov test. Continuous variables were analysed with independent samples t-test or Mann–Whitney U-test as appropriate. Categoric variables were compared with Chi-squared tests. The frequency of missing responses was analysed in all questions and its difference across the two study groups was compared with Chi-square tests. Statistical significance (*p* < 0.05) was considered only in variables without significant differences in the distribution of missing responses. Since in independent component multivariate analysis, finding the most influential driver factors are critical, we applied the stepwise logistic regression to find potential predictive models for subjective memory complaints with the inclusion of the 20 analysed variables (predictor variables)^26,27^. Where participants preferred not to say, responses were considered as missing variables. The response variable was set as the grouping variable (SCC). Predictor variables were continuous variables (age, educational attainment, BMI), categoric variables (sex) or directly generated as categorical variables from scale-based answers. Significance of p was set at  < 0.05. Results of logistic regression were reported with significance levels, odds ratios (ORs) and 95% confidence intervals (CIs).

## Results

### Sociodemographic factors

The SCC+ cohort was significantly older than the SCC− (MD = 3.6 years; *p* < 0.001) and there were more women in the SCC+ group (Chi = 21.1; *p* < 0.001). The missing response rate was not statistically different in the above mentioned two parameters. The average educational years in the SCC+ cohort was higher than in the SCC− but the difference was non-significant. The “married” marital status was the most common in both measured groups, but there were more married participants in the SCC− than in the SCC+ group (54.2% vs. 44.6%). Parallel to this, the divorced (10.8% vs. 7.2%) and the widowed (30.1% vs. 25.4%) subjects were overrepresented in the SCC+ population. Pensioner was the most common work status in the population (72.1% in SCC− and 72.6% in SCC+ groups), while 15.5% of the subjects in the SCC− and 18.6% in the SCC+ group were working after retirement. Only 8% of the whole study population was actively working. Statistical results of between group comparisons are presented in Table 2.

**Table 2:** Characteristics of the responder population.

Parameter	SCC−	Prefer not to say (%)^b^	SCC+	Prefer not to say (%)^b^	*p*-value (effect size in Cohen’s d)
Number of participants	271	–	88	–	–
Sociodemographic factors					
^a^Age (years)	**72.6 ± 7.4**	0%	**76.2 ± 8.9**	0%	1
					< 0.001* (**0.44**)
^b^Sex (% of females)	**61%**	0%	**65%**	0%	0
					< 0.001* (**0.24**)
^c^Educational attainment (years)	14.5 (12–17)	2.50%	15 (12–17)	4.50%	< 0.001*
					0.66
^b,d^Family status					< 0.001*
-Single (%)	7.95%	1.80%	8.43%	4.50%	0.631
-Married (%)	54.17%		44.58%		< 0.001*
-Living with partner (%)	3.41%		6.02%		< 0.001*
-In relationship, living separately (%)	1.89%		0%		< 0.001*
-Divorced (%)	7.20%		10.84%		< 0.001*
-Widowed (%)	25.38%		30.12%		< 0.001*
^b,e^Work status					< 0.001*
-Employed (%)	7.43%	0.70%	7.14%	4.50%	< 0.001*
-Temporally unemployed due to pandemic (%)	0.37%		1.19%		< 0.001*
-Unemployed (%)	0.74%		1.19%		< 0.001*
-Pensioner (%)	72.12%		72.62%		< 0.001*
-Working as a pensioner (%)	18.59%		15.48%		< 0.001*
-Invalid (%)	0.37%		2.38%		< 0.001*
Prepandemic physical condition and lifestyle					
^b,f^Prepandemic smoking					< 0.001*
-No (%)	88.56%	0%	85.06%	1.10%	< 0.001*
-Occasionally (%)	2.58%		2.30%		< 0.001*
-Daily (%)	8.86%		12.64%		< 0.001*
^c^Prepandemic number of chronic diseases (number)	**2 **(**1–3**)	0.59%	**2 **(**1–4**)	0.55%	0.67 *p* < 0.001* (**0.46**)
^c^Prepandemic BMI (number)	26.6 (24–29.7)	2.90%	27.4 (24–30.6)	1.10%	< 0.001* 0.35
^b,g^Prepandemic alcohol consumption (international unit/day)					< 0.001*
-1–2 (%)	80.43%	16.20%	86.21%	34%	< 0.001*
-3–4 (%)	16.85%		6.90%		< 0.001*
-5–6 (%)	1.63%		5.17%		< 0.001*
-7–9 (%)	1.09%		0%		< 0.001*
- > 10 (%)	0%		1.72%		< 0.001*
^b,h^Physical mobility (walking 500 m independently)					0.29
-Easily able (%)	**86.89%**	1.15%	**58.62%**	1.10%	< 0.001* (**0.36**)
-Able but with difficulties (%)	**7.12%**		**26.44%**		< 0.001* (**0.89**)
-Barely able (%)	**3%**		**4.60%**		< 0.001* (**0.37**)
-Not able (%)	**3%**		**10.34%**		< 0.001* (**0.87**)
Changes in lifestyle during the pandemic					
^b,i^Time for family					< 0.001*
-Decreased (%)	79.06%	12.90%	77.92%	12.50%	< 0.001*
-Same (%)	13.68%		15.58%		< 0.001*
-Increased (%)	7.26%		6.49%		< 0.001*
^b,i^High quality sleep time					< 0.001*
-Decreased (%)	33.13%	38.70%	70.49%	30.60%	< 0.001*
-Same (%)	63.86%		27.87%		0.0207*
-Increased (%)	3.01%		1.64%		0.04*
^b,i^Time for physical activity		19.90%		20.40%	< 0.001*
-Decreased (%)	41.01%		52.86%		< 0.001*
-Same (%)	42.86%		35.71%		0.003*
-Increased (%)	16.13%		11.43%		0.039*
^b,i^Time for remote work					< 0.001*
-Decreased (%)	1.72%	78.50%	0%	85.20%	0.192
-Same (%)	44.83%		23.08%		0.094
Increased (%)	53.45%		76.92%		< 0.001*
^b,i^Time for internet use					0.91
-Decreased (%)	**1.96%**	24.70%	**0%**	24.60%	0.001* (**0.47**)
-Same (%)	**42.16%**		**24.70%**		< 0.001* (**0.42**)
-Increased (%)	**55.88%**		**75.93%**		< 0.001* (**0.32**)
Changes in social engagement during the pandemic					
^b,j^Time for grandchildren					< 0.001*
-Decreased (%)	33.58%	9.10%	42.05%	10.10%	< 0.001*
-Same (%)	62.73%		54.55%		< 0.001*
-Increased (%)	3.69%		3.41%		< 0.001*
^b,j^Time for voluntary work					< 0.001*
-Decreased (%)	6.27%	10.30%	9.09%	10.90%	< 0.001*
-Same (%)	91.51%		88.64%		< 0.001*
-Increased (%)	2.21%		2.27%		< 0.001*
^b,j^Time for educational activity					< 0.001*
-Decreased (%)	8.49%	7.60%	7.95%	6.60%	< 0.001*
-Same (%)	90.41%		88.64%		< 0.001*
-Increased (%)	1.11%		3.41%		< 0.001*
^b,j^Time for sport and social clubs					< 0.001*
-Decreased (%)	20.3%	13.40%	12.50%	11.90%	< 0.001*
-Same (%)	78.23%		86.36%		< 0.001*
-Increased (%)	1.48%		1.14%		< 0.001*
^b,j^Time for patient organizations					< 0.001*
-Decreased (%)	9.59%	16.30%	13.64%	12.80%	< 0.001*
-Same (%)	89.67%		84.09%		< 0.001*
-Increased (%)	0.74%		2.27%		< 0.001*

As data demonstrates, there was a large variety among the missing responses between the two study groups. Intergroup differences were considered relevant only in the cases where groups did not differ significantly in the missing response rate (*p* > 0.05). Bold signalling indicates the statistically significant differences between the study populations with the consideration of the above-mentioned circumstances. Effect size is measured in Cohen’s d in the variable category with significant differences, where 0.2–0.5 = small effect, 0.5–0.8 = medium effect,  > 0.8 = large effect. As the Table indicates, in the differences in physical independence, even large effect sizes are observed, while in the rest of the parameters, small effect sizes are presented.

Key: SCC subjective cognitive complaints, BMI body mass index.

^a^Defined in mean ± SD. Statistically compared with t-test, where * indicates significant p (< 0.05).

^b^Defined in %. Statistically compared with Chi-square test, where * indicates significant p (< 0.05).

^c^Defined in median (interquartile range). Statistically compared with Mann–Whitney U- test, where * indicates significant* p* (< 0.05).

^d^Possible answers- 1: single, 2: married, 3: living with partner, 4: in relationship, living separately, 5: divorced, 6: widowed, 7: prefer not say.

^e^Possible answers- 1: employed, 2: temporally unemployed due to pandemic, 3: unemployed, 4: pensioner, 5: working as a pensioner, 6: prefer not say.

^f^Possible answers- 1: no, 2: occasionally 3: daily.

^g^Possible answers- 1: 1–2 international unit (IU), 2: 3–4 IU, 3: 5–6 IU, 4: 7–9 IU, 5: > 10 IU/day).

^h^Possible answers for ability to walk 500 m independently- 1: easily able, 2: able but with difficulties, 3: barely able, 4: not able.

^i^Possible answers- 1: significantly decreased, 2: decreased, 3: same, 4: increased, 5: significantly increased.

^j^Possible answers- 1: daily, 2: few times per week, 3: once per week, 4: few times per month, 5: once per month, 6: less than once per month, 7: never. Pre- and postpandemic responses are highlighted on a scale ranging from − 6 (maximum increase) to + 6 (maximum decrease).

### Pre-pandemic physical condition

While the majority of the participants were non-smoker (88.6% in the SCC− and 85.1% in the SCC+ group), daily smoking was higher in the SCC+ population (12.6% vs. 8.9%). Most of the responders were mild drinkers consuming 1–2 international units/day (80.4% in the SCC− and 86.21% in the SCC+ group). Moderate alcohol consumption (3–4 IU/day) was more common in the SCC− group (16.9% vs. 6.9%). People in both groups were characterized by mild overweight with an average BMI of 26.6 in the SCC− and 27.4 in the SCC+ group. Chronic disorders were frequently reported: only 16.7% of the SCC− and 6.9% of the SCC+ patients indicated the absence of any chronic condition. Fifty-eight percent of SCC− subjects had at least 2 chronic disorders and it was even more frequent in the SCC+ group (76.4%). Based on these data, the measured population was characterized by polymorbid chronic medical conditions; however, the number of concomitant disorders was significantly higher in the SCC+ population (U = 8354.5; *p* < 0.001). This might be in line with the physical mobility measurements, since less patients reported the ability to walk 500 m without difficulties in the SCC+ than in the SCC− group (58.6% vs .86.9%). Ten percent of the SCC+ participants were not able to walk independently, and it was less characteristic in the SCC− group (3%). The reported difference was significant. The missing response rate was not statistically different in physical independence and the number of chronic illness categories. The results of between group comparison statistics are presented in Table 2.

### Changes in lifestyle

The time dedicated to family was greatly reduced in both groups, 78% of the participants irrespective of which group they were in, reported significant reduction, while only 7% indicated increase in this activity. The frequency of physical activity also decreased in a large proportion of subjects (significant reduction was detected in 20% of the SCC+ and in 11.5% of the SCC− group, while slight reduction was present in 32.9% of the SCC+ and 29.5% of the SCC− patients). Only 13% reported at least slightly increased sport activities without significant intergroup differences. Sleep problems were more frequent in the SCC+ than in the SCC− group (70.5% vs. 33.1% respectively). Improvement in subjective sleep quality was reported by less than 3% of the responders. Remote working was increased in 76.9% of SCC+ and 53.4% of SCC− subjects. Reduction was indicated in less than 2% of the participants. While SCC+ patients showed higher decrease in physical activity and increase in sleep problems and remote working, compared to SCC− subjects, the only parameter without dominant missing response differences is the internet use. Seventy-six percent of SCC+ patients versus 55.9% of the SCC− patients reported more intensive use with statistical significance (*p*’s < 0.001). Furthermore, constant internet use was demonstrated less frequently in the SCC+ group (24.7% vs. 42.2%). Statistical results of between group comparisons are presented in Table 2.

### Changes in social engagement

Most of responders spent less time with grandchildren. Consistency was less frequently reported in the SCC+ group (54.6% vs. 62.7%). A 1-point decrease was the most common in both groups (15.9% in the SCC+ and 12.6% in the SCC− group); however, a large 4-point decrease was also frequently found among the SCC+ population (10.23% in SCC+ vs. 3.3% in SCC− group). Changes in voluntary work (11%) and educational activities (10%) were barely reported. A slight reduction was visible in the time spent in social and sport clubs as 17% reported reduction (3.2-point change in average). Only 10.5% of responders indicated less time in patient organizations, but the change was mild (1.1-point change in average). Significant missing response differences were presented between the study groups in all categories. Statistical results of between group comparisons are presented in Table 2.

### Factors associated with SCC

**Figure 2:**
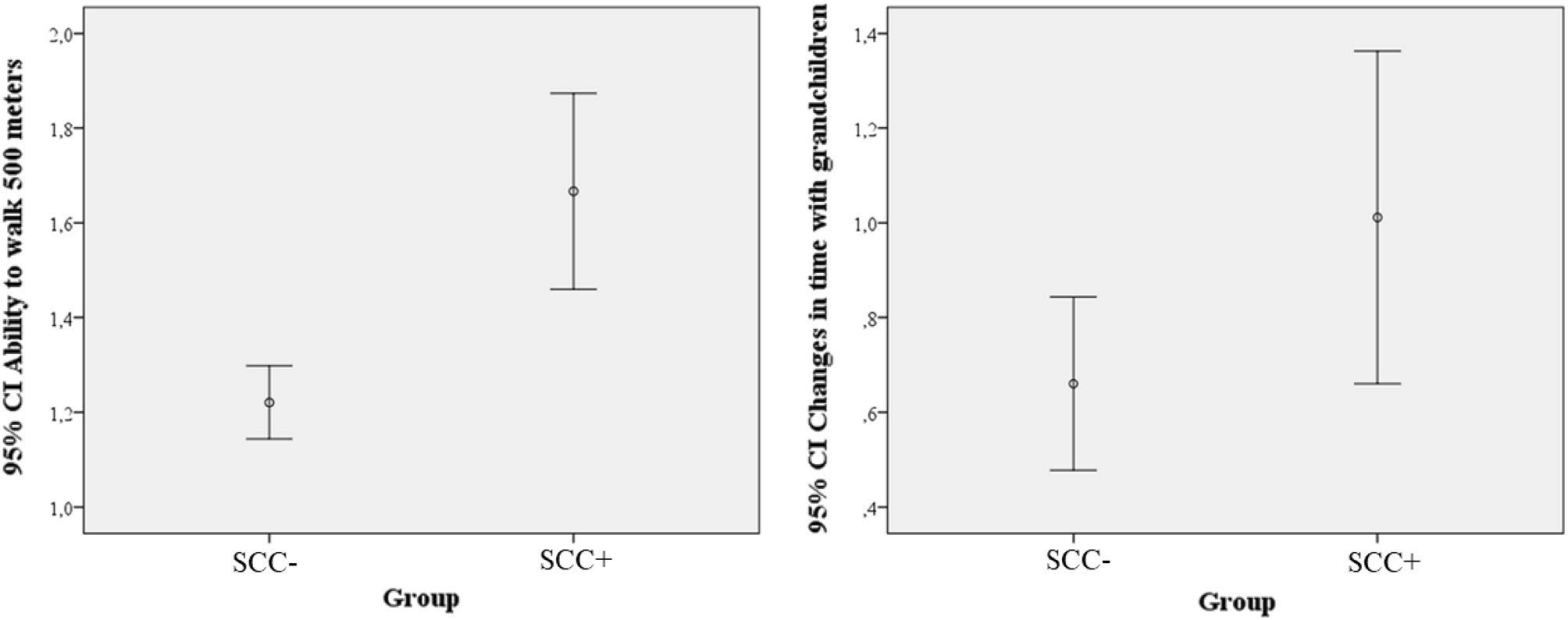
Differences in physical mobility and time with family across healthy participants and responders developing subjective cognitive complaints (SCC) during the 3rd wave of COVID pandemic in Hungary. Logistic regression revealed that the key parameter indicating the development of SCC is the reduced physical mobility. Physical mobility was assessed on a 4-point scale where higher scale indicates poor performance. The second important contributor of SCC in our sample is the time spent with grandchildren. Responses were compared on a 7-point scale assessing the estimated time before and during the pandemic. Positive change indicates less time with family. Key: SCC subjective cognitive complaints.

Stepwise logistic regression was used to analyse the factors associated with SCC (Table S2 in Supplementary Material). The applied model highlighted that only two parameters defined the outcome of the responders, the physical mobility and independence (ability to walk 500 m without difficulties; OR = 1.186; *p* < 0.001; 95%CI = 1.101, 1.270) and changes in time spent with grandchildren (OR = 1.04; *p* = 0.015; 95%CI = 1.008, 1.073). The reported model included only the physical mobility reached 0.082 R square, while the model including both parameters reached 0.108 R square. The improvement of R square values between the two models was significant (*p* = 0.026). Intergroup differences are presented with error bars in Fig. 2.

## Discussion

In this study, after exclusion, we analysed the data of 359 elderly Hungarians who filled out the WW-Fingers SARS-CoV2 survey. The majority of questionnaires (83%) were completed in February and March 2021, in the first part of the third wave of COVID-19 pandemic in Hungary. Our participants typically live in the capital city and its suburban area, they have a higher-than-average level of education, are mostly retired, and have more than one chronic disease. An important observation was that a quarter of the respondents (n:88) reported subjective cognitive complaints affecting their memory functions that could be related to the COVID-19 pandemic. Our goal was to analyse the sociodemographic features, health status, lifestyle, and social life parameters (altogether 20 features) to characterize the most significant contributors associated with SCC within our cohort. Noticeably, the two measured groups demonstrated significant differences in many parameters regarding the number of missing responses, making the data analysis more complicated. However, participants with SCC showed special characteristics compared to individuals without SCC: (1) they were older; (2) they were more likely to be women; (3) they had on average a higher number of chronic disorders; (4) showed more prominent impairment in physical mobility; (5) had worse sleep quality; (6) spent less time with family; and (7) used internet more frequently during the pandemic (all *p*’s < 0.001). To eliminate the potential interrelation across these group differences, stepwise logistic regression was applied. As a major finding of this model, impaired pre-pandemic physical mobility and reduced time spent with family during the pandemic were the most characteristic associated with SCC.

Noticeably, even though no memory tests have been carried out, our cohort represented an SCD plus category since: (1) they reported dominantly memory impairment; (2) symptoms developed within the last 5 years; (3) the age of the responders was  > 60 years^21^. The analysed population lends special importance to our findings in the light of studies showing that SCD plus individuals have the highest risk to convert into MCI^21^.

Our results show that older age is associated with increased likelihood of SCC. This is not surprising, as several studies have confirmed an aging-related increase in the incidence of dementia and MCI^28^. The higher likelihood for the development of cognitive complaints in women is also in line with the current literature. Recent evidence shows that women have a higher chance to show cognitive symptoms in the presence of Alzheimer brain pathology even in the preclinical stages^29,30^; they show higher conversion from MCI to AD^31^; and have a higher risk for AD^32^. In our analysis, marital status, education, and labour market position were not associated with the presence of SCC; however, our data acquisition follows a special set-up making generalization of these features complicated due to the significant differences between the study groups in regard of the missing responses. Impact of work status is barely measurable in our cohort since the age  > 60 years was an inclusion criterion. While low educational years associate with higher dementia risk^33^, a possible explanation for our results is the high level of education in the entire subject population. Some results reported that living alone increases the risk for SCC^34^ and for dementia^35^. We distinguished numerous marital status categories which might decrease the discriminative effect compared to other studies using only strict grouping variables (living with partner or without). However, a trend was also visible in our sample showing higher proportion of divorced and widowed status in SCC+ group. Furthermore, the protective effect of family was clearly reinforced by the regression analysis pointing to the important of time spent with grandchildren as the second most important predictor of SCC.

In the sample, the number of people living with chronic diseases was remarkably high, affecting almost 90% of the respondents, most of them being diagnosed with 2 diseases. Presence of SCC was associated with higher number of concomitant chronic disorders. Since it is well known that parallel to the increase in the number of chronic diseases, the patient's immobility and vulnerability also increases in the majority of the cases, several studies have confirmed their role in the development of SCC^36–38^. Although, in order to prevent dementia, the classic cardiovascular risk factors should be prevented^23^. Probably the effect of alcohol and smoking is non-measurable since both populations reported only a low dose of alcohol consumption (mild drinkers) and relatively low frequency of smoking. Even if not confirmed by further statistical analyses, it is important to note that 34% of SCC+ (compared to 16% of SCC−) refused to report drinking alcohol before the epidemic, and of those who did report, nearly 7% of SCC+ drank heavily before the epidemic. SCC+ patients also smoked more than SCC− (12.6% vs. 8.9%). Both factors are much studied and have a significant role in the development and progression of cognitive decline^39,40^. Reduced physical activity and immobility is considered nowadays as one of the most important risk factors of cognitive deterioration^24,41,42^ and our results also highlighted its crucial and leading predictive value in the development of SCC.

In our study, among the changes in lifestyle factors, the increase in internet use should be highlighted. More than 60% of respondents reported an increase in internet usage and daily use of digital devices. Increased internet use can reduce time spent on physical activities, which can cause anxiety^43^ and increase feelings of loneliness^44^, each of these factors may have been independently significantly affected by pandemic-related restrictions. Based on our data, the more frequent use of digital services did not lead to the strengthening of social relations, even though this method of contact would have made it possible. Another important point is that for the elderly, increased digital living space can reduce isolation^45^, but increased use results in a drastic decrease in satisfaction in this population based on large samples^46^. Based on previous research data, increased internet use is often accompanied by reduced sleep time, later bedtimes and earlier waking up^47^. This factor is also significant in our research, since sleep disorders became more pronounced in almost half of the respondents, which was found in several studies to be related to the deterioration of subjective memory^48–50^. One of the most promiment differences between the groups is how greatly the sleep quality decreased in SCC+ vs. SCC−. Sleep disorders may be a prodrome to dementia and sleep has an active role in information processing, with non-rapid eye movement (NREM) being predominantly responsible for processing declarative content and rapid eye movement (REM) sleep for processing non-declarative content^51,52^. During NREM sleep, acquired information is reactivated and integrated into long-term memory, which is stabilized by a synaptic consolidation process during REM sleep^53^. Furthermore, higher frequency of sleep disorders and impaired sleep quality in relation to pandemic was observed with the same survey in the United Kingdom in 37% of the responders^20^, so the increase in sleep problems seems to be general across the various geographical regions.

Based on our results, it can be concluded that although the use of digital services has increased significantly, the time spent with family and close friends has decreased drastically. Among the social activities, volunteering, participation in education, sports, visiting social and other clubs were characteristic in only small ratio of the respondents, even in the period before the pandemic. Furthermore, the ratio of missing responses in social engagement characteristic showed large variety across the study groups. It should be noted that the low engagement in these activities in the Hungarian population might have a negative effect in general, since these are well-known preventive factors of cognitive functions^24^. The most significant predictive factor in the development of subjective cognitive complaints is the decrease in the frequency of meetings with grandchildren. The lack of that as a source of joy can also greatly affect the emotional life. Almost 80% of the respondents reported that the time spent with family members and friends decreased significantly. This is in line with observations using the same survey in other countries: 55% of the participants reported less contact with friends and 33% with family in a Finnish observation^18^. The connection between social isolation and the increase in the number of affective disorders was also described as a possible indirect effect of the Covid-19 pandemic^54^ and it should be carefully considered as a possible long-term change in social behaviour.

In our sample, after performing the regression analysis, two clear risk factors could be highlighted: (1) Physical immobility is the greatest risk and we need to pay special attention to the patient population with low mobility to lessen dementia risk following the pandemic; (2) The time spent with grandchildren is the second most dominant predictor for developing SCC. Reduced physical activity and immobility are currently regarded as a significant risk factor of cognitive deterioration^24,41,42^. The role of family and social contacts in the prevention and the progression of cognitive decline has long been investigated^55,56^. These findings highlight the importance of physical activity and close social relationships as a key aspect of healthy brain ageing that is usually overlooked, although many studies have demonstrated their importance^24,57,58^. This must be also considered on a broad social level since pandemics might change our social relationships on a more consistent way than the short-term restrictions.

To our knowledge, this study was the first in our region that examines the factors predisposing to subjective memory disorder in a larger number of cases. In addition, our research has several important limiting factors. Firstly, due to the characteristics of the international questionnaire, a self-completion cross-sectional test was used, health data on the participants' previous physical and mental health status were only partially known and their assessment was based on the subjective opinion of the respondent. Our results may have been influenced by the fact that some respondents may have been unaware of their COVID-19 infection or denied it. We could not control the changes in the living situation, these were also self-reported changes. It can be assumed that in most cases these changes were caused by the pandemic and related restrictions. The data collection follows a specific design, which makes it difficult to generalise these characteristics. No memory screening test was performed. Secondly, the sample reflects a specific, highly educated, well-cooperating population. Due to the uncertainty surrounding the pandemic, our survey was not representative, we used convenience sampling. Our results are based primarily on data from people living in the Hungarian capital or its suburbs and are therefore not generalisable. Finally, the large variety in the missing responses in numerous parameters complicate the comparisons. This fact also draws attention to a common problem of dementia research analysing the impact of sociodemographic factors. However, it can be assumed that in terms of the main findings, similar changes can be expected in populations belonging to other geographical areas, although their extent cannot be estimated based on the present data. Further joint-analyses are needed within the framework of the World-Wide Finger network.

### Supplementary Information

Supplementary Tables.

## Data Availability

Raw data are available upon request to the corresponding author.
